# MAS-NMR of [Pyr_13_][Tf_2_N] and [Pyr_16_][Tf_2_N] Ionic Liquids Confined to Carbon Black: Insights and Pitfalls

**DOI:** 10.3390/molecules26216690

**Published:** 2021-11-05

**Authors:** Steffen Merz, Jie Wang, Petrik Galvosas, Josef Granwehr

**Affiliations:** 1 Fundamental Electrochemistry (IEK-9), Institute of Energy and Climate Research, Forschungszentrum Juelich, 52425 Juelich, Germany; s.merz@fz-juelich.de (S.M.); j.granwehr@fz-juelich.de (J.G.); 2 MacDiamid Institute for Advanced Materials and Nanotechnology, School of Chemical and Physical Sciences, Victoria University Wellington, Wellington 6140, New Zealand; jie.wang@vuw.ac.nz; 3 Institute of Technical and Macromolecular Chemistry, RWTH Aachen University, 52056 Aachen, Germany

**Keywords:** [Pyr_13_][Tf_2_N], [Pyr_16_][Tf_2_N], MAS, CPMG, 13-interval PGSTE, VXC72 carbon black, diffusion-NMR, Ionic liquids

## Abstract

Electrolytes based on ionic liquids (IL) are promising candidates to replace traditional liquid electrolytes in electrochemical systems, particularly in combination with carbon-based porous electrodes. Insight into the dynamics of such systems is imperative for tailoring electrochemical performance. In this work, 1-Methyl-1-propylpyrrolidinium bis(trifluoromethylsulfonyl)imide and 1-Hexyl-1-methylpyrrolidinium bis(trifluoromethylsulfonyl)imide were studied in a carbon black (CB) host using spectrally resolved Carr-Purcell-Meiboom-Gill (CPMG) and 13-interval Pulsed Field Gradient Stimulated Echo (PFGSTE) Magic Angle Spinning Nuclear Magnetic Resonance (MAS-NMR). Data were processed using a sensitivity weighted Laplace inversion algorithm without non-negativity constraint. Previously found relations between the alkyl length and the aggregation behavior of pyrrolidinium-based cations were confirmed and characterized in more detail. For the IL in CB, a different aggregation behavior was found compared to the neat IL, adding the surface of a porous electrode as an additional parameter for the optimization of IL-based electrolytes. Finally, the suitability of MAS was assessed and critically discussed for investigations of this class of samples.

## 1. Introduction

The investigation of ionic mobility plays a crucial role for the understanding and quantification of electrochemical systems such as batteries, fuel cells, or electrolyzers [1]. Important parameters include transference numbers, diffusion, and aggregation in liquid-and ionic liquid-based electrolytes or ion mobility in ion-conducting solid electrolytes [2]. The investigation of species inside porous matrices, which are of importance in metal-air batteries, or in solid ceramic or hybrid ceramic-polymer electrolytes for solid-state batteries is particularly challenging [3]. In both cases, ions move in different environments or across interfaces; hence, multiple environments must be distinguished to obtain a comprehensive description of dynamic processes.

Nuclear magnetic resonance (NMR) is exquisitely sensitive to the structure, environment, and dynamics of nuclei with non-zero nuclear spin [4,5,6,7,8,9]. Generally speaking, spectroscopic information provides structural and electronic information about the immediate molecular surroundings of a nucleus, while relaxation measurements describe local dynamics over a range of time scales, averaged over milliseconds to seconds. Slow processes can further be characterized using exchange experiments, which allow the identification of exchanging species and their exchange time constants [10,11]. Mobility on length scales of µm can be quantified using complementary pulsed field gradient (PFG) NMR experiments [12,13]. Solid samples show anisotropic spin interactions, which can be averaged out by magic angle spinning (MAS) [14,15]. Equally, line broadening caused by differences of magnetic susceptibility between materials in multi-component samples, such as liquids in porous media, can be reduced by MAS. The combination of MAS and PFG would be very attractive to retain the high spectral resolution for the identification of different species and their immediate environment and to correlate this information with long-range mobility in a similar way, as it was established for liquids and homogeneous liquid mixtures with the diffusion ordered spectroscopy (DOSY) experiment [16,17]. However, the combination of MAS and PFG NMR is not yet routinely employed.

Electrochemical systems are heterogeneous by nature, containing multiple components with potentially different states of matter. The study of ionic mobility in electrochemical systems would clearly benefit from a combination of MAS and PFG; however, the involved sample preparation and experimental protocols combined with limited access to, and limitations of, suitable hardware have prevented its wider application so far. However, careful experiment design may obviate these challenges. Limitations may include, in some cases, the minimum number of spins required; but experimental challenges may not be limited to this aspect [18]. In fact, electrochemical systems often contain ions in high concentrations, which imply sufficient sensitivity. On the other hand, solid-state samples and liquids in small pores or at interfaces often show short *T*_2_ relaxation time constants, which may place constraints on the available time for applying pulsed field gradients. This can only be compensated by increased gradient amplitudes within given hardware capabilities, beyond which the method will fail. Furthermore, heterogeneous samples may be altered by fast spinning, either due to centrifugation or mechanical stress [19]. Therefore, care has to be taken because various parameters of the measuring system, e.g., spinning speed, gradient strength, or rotor filling, can become major sources of errors or influence the results due to altered sample structure. To avoid running long experiments that are eventually futile, the experimental constraints must be derived in advance from reference experiments, mapping out the available parameter space.

Carbon black (CB) is the generic name for a variety of finely divided carbon pigments originating from either pyrolysis or incomplete thermal decomposition of hydrocarbons under inert or oxygen-depleted atmosphere [20,21]. During the process, particles agglomerate to turbostratic layers. These units condense to stacks, which contain edge atoms that are reactive and adhere into chain-like structures [22]. Despite possessing roughly the same parallel and equidistant stacks of layer planes as graphite, these kinds of carbon lack a three-dimensional ordering on larger scales. In general, CB is a good electron conductor and is, therefore, commonly employed for various electrochemical applications including fuel cells [23,24], supercapacitors [25], sensors [26,27], or batteries [28,29], e.g., as additive in lithium-ion batteries (LIB) [30,31] or for gas diffusion electrodes in metal-air batteries [32]. 

Ionic liquids (IL) are Coulomb fluids entirely comprised of ions, with a melting point, by definition, below 373 K. They exhibit a higher conductivity than organic or aqueous electrolytes, are able to withstand voltages up to 5 V, offer large thermal and chemical stability, and possess a negligible vapor pressure [33]. ILs can be tailored by combining different anions and cations to meet individual requirements; hence, they are intensively investigated as possible replacements for aqueous electrolytes and are employed for a wide range of electrochemical applications [34,35,36]. In ILs, polarization, π-π, dipole-dipole, solvophobic, and van der Waals interactions (short-range) act together with Coulombic electrostatic forces (long-range). These inter- and intramolecular interdependencies can cause nanostructuring and complex IL dynamics in bulk, at surfaces, and under confinement [37,38,39]. Furthermore, since strong electrostatic interactions influence the dynamics, ILs behave distinctly differently than molecular liquids. 

If confined to carbon matrices, the ordering of ILs depends mostly on the chemical structure and morphology of the confining matrix, its pore size distribution, and on the size of the IL anions and cations [40,41,42,43]. It has been demonstrated that a certain lateral ordering of an IL develops, which, e.g., enhances lubricity by forming crystal-like structures on ionophilic surfaces, depending on the polarity of the matrix [44,45,46]. This has also been shown for carbon surfaces under an applied potential [40,47,48]. 

ILs based on pyrrolidinium cations (Pyr) have the potential to outperform organic electrolytes and possess the advantage that the length of the alkyl chain can be used to adapt the properties of the neat IL [49,50]. Depending on the length, *n*, of the alkyl chain, this class of ILs can be split into short-chain (1 ≤ *n* ≤ 4) and long-chain (*n* ≥ 5) subgroups. The latter is characterized by weaker ionic interactions. For side chains with *n* ≥ 4, the ILs form nanostructures and segregate into nonpolar domains driven by van der Waals interactions [51]. The anions and the aromatic part of the cations of these domains can form a 3D network of ionic channels and facilitate distinct nano-domains, which lead to dynamic heterogeneities [52,53,54]. In NMR, transverse relaxation time constants (*T*_2_) are strongly influenced by entropic changes, e.g., molecular structuring and correspondingly altered dynamics, whereas diffusion measurements are particularly sensitive regarding the clustering of ILs. Furthermore, external electric and magnetic fields can change the configuration of aggregates [55,56,57,58].

In an earlier study, we investigated the dynamics of 1-Methyl-1-propylpyrrolidiniumbis(trifluoromethylsulfonyl)imide ([Pyr_13_][Tf_2_N]) confined to CB using PGSTE-NMR in combination with *T*_1_ relaxation time measurements [59]. We found evidence for a quasi-stationary IL film coating the carbon surface with a lubricating effect on the layer directly above. This layer showed an increased long-range mobility, causing an increased overall motion along the carbon surface [59]. However, identification of different environments using variations of their spectral signature was impeded by low resolution due to susceptibility variations inside the porous carbon environment or by incompletely averaged anisotropic interactions [60]. Such a line-broadening effect could be mitigated by using MAS [61]. 

In this paper, we are investigating the suitability and limitations of PFG NMR in combination with MAS for the study of ionic mobility in materials for electrochemical applications. While both solid superionic lithium conductors and electrolytes in porous electrode materials are of interest, the focus here is on the latter, as it exhibits somewhat reduced demands on the hardware in terms of pulse length or gradient strength, allowing for a more sensitive characterization of limitations. Nonetheless, the results can also serve as a feasibility assessment of experiments on solid electrolytes. The effects of confinement to CB on the dynamics of [Pyr_1*n*_][Tf_2_N] with different alkyl side chain lengths of *n* = 3 and *n* = 6 were investigated. We used a 13-interval stimulated echo PFG method and *T*_2_ measurements in combination with MAS in order to establish a protocol to describe the behavior of IL confined to a CB matrix. We further report on certain pitfalls associated with MAS NMR, e.g., the electrical conductivity of CB under pressure [62,63,64]. However, an exhaustive interpretation of interactions between ILs and CB is outside the scope of this paper since a much larger set of systematic experiments in combination with numerical simulations would be required.

## 2. Results and Discussion

### 2.1. IL Loading Dependence of ^1^H NMR Spectra

**Figure 1:**
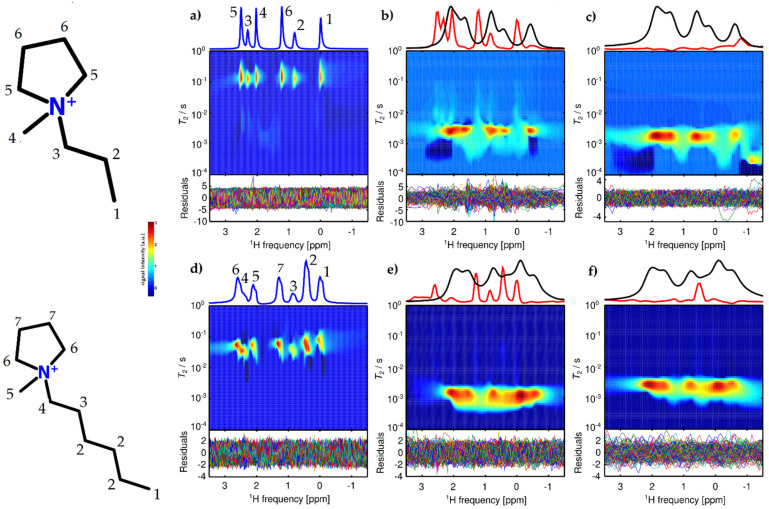
Pseudocolor plot of [Pyr_13_] (**a**–**c**) and [Pyr_16_] (**d**–**f**) *T*_2,app_ relaxation time distribution vs. ^1^H NMR frequency: (**a**) neat [Pyr_13_][Tf_2_N], (**b**) sample with CB and S_IL_ = 0.2 of [Pyr_13_][Tf_2_N], (**c**) sample with CB and S_IL_ = 1 of [Pyr_13_][Tf_2_N], (**d**) neat [Pyr_16_][Tf_2_N], (**e**) sample with CB and S_IL_ = 0.2 of [Pyr_16_][Tf_2_N], (**f**) sample with CB and S_IL_ = 1 [Pyr_16_][Tf_2_N]. The colormap is scaled with the square root of the amplitude to amplify features with low intensity. At the top of the panels of the neat IL, the sum projection onto the frequency axis is shown (blue). At the top of the IL in CB panels, the integral of the main feature (black; integrated from 1.3 ms to 5.0 ms for (**b**), from 0.36 ms to 2.5 ms for (**c**), from 0.7 ms to 3.2 ms for (**e**), and from 0.9 ms to 4.5 ms for (**f**)) and the integral of the slowly relaxing region (red; integrated from 5.6 ms to 180 ms for (**b**), from 4.0 ms to 100 ms for (**c**), from 3.6 ms to 56 ms for (**e**), and from 5.6 ms to 320 ms for (**f**)) are shown. For the [Pyr_13_][Tf_2_N] samples, the red spectrum is scaled by a factor of 15 compared to the black spectrum. For [Pyr_16_][Tf_2_N], the red spectrum is scaled by a factor of 100 compared to the black spectrum. Since the narrow signal represented by the red spectrum extends into the main relaxation mode, the signal intensity of the red species represents a lower limit in terms of contributing spins. The weighted residuals are shown at the bottom of each panel.

Figure 1 shows spectrally resolved maps of apparent ^1^H transverse relaxation time constants, *T*_2,app_, for [Pyr_13_][Tf_2_N] (Figure 1a) and [Pyr_16_][Tf_2_N] (Figure 1d) as neat ILs and loaded into the pore space of CB (Figure 1b,c,e,f), which was filled at two different fractions. NMR measurements of the neat ILs were recorded statically (no MAS spinning), while the IL-loaded CB samples were measured under MAS at 5 kHz. 

The ^1^H NMR spectra of the neat ILs showed chemical shift resolution of the different resonances, yet the resolution was not sufficient to also resolve J-couplings. While for [Pyr_16_][Tf_2_N] the lack of resolution was consistent with the measured *T*_2,app_, for [Pyr_13_][Tf_2_N], a distribution of chemical shifts seemed to additionally cause a somewhat asymmetric broadening. [Pyr_16_][Tf_2_N] did not show such an asymmetry, but two sets of spectra with similar intensities were observed, both of which were clearly caused by the same IL but shifted by about 0.07 ppm with respect to each other.

Confining both ILs to CB caused a broadening of all individual spectral features, driven by susceptibility differences between different phases, by local ring currents at the carbon surface, and by local magnetic fields induced by the electrically conductive matrix. This behavior is in line with the dynamics of molecular liquids in porous media [65,66]. Under confinement for small IL-loading fractions (Figure 1b,e) the main feature of the spectra of [Pyr_13_][Tf_2_N] and [Pyr_16_][Tf_2_N] shifted ≈ 0.5 ppm upfield. These shifts were primarily caused by ring current effects of the aromatic groups on the surface of the carbon matrix [67]. For the CB samples with *S*_IL_ = 1 (Figure 1c,f), the spectra of the ILs shifted upfield by approximately 0.4 ppm and 0.6 ppm for [Pyr_13_][Tf_2_N] and [Pyr_16_][Tf_2_N], respectively.

The samples with S_IL_ = 0.2 loading of the CB pore space also showed additional spectral features compared to the samples with high IL loading for both ILs. At low pore space loading, a less rapidly relaxing second species with a spectrum very similar to the neat IL, showing a considerably broadened *T*_2,app_, could be observed. The spectra, integrated along the relaxation time dimension, are shown in the top panels of Figure 1 b,c,e,f. Since the position of the main relaxation component varied for the investigated samples, the integration limits were individually adjusted (see caption of Figure 1). Despite its low intensity, this feature could be clearly identified due to the chemical shift difference and the increased resolution afforded by MAS. The absence of such a separate narrow component for a sample with its pore space fully loaded is consistent with a previous report on MAS NMR of a similar system with a carbonaceous matrix fully saturated with [Pyr_13_][Tf_2_N] [61].

### 2.2. ^1^H-T_2,app_ Time Distributions

The *T*_2,app_ distribution of the neat [Pyr_13_][Tf_2_N] was dominated by one mode with a mean relaxation time constant of 155 ms. All peaks showed a *T*_2,app_ in the range between 130 ms to 160 ms, where all distributions showed a weak trend towards shorter *T*_2,app_ for the broadened upfield side of each line. In addition, weak features were visible at a very short relaxation time below about 10 ms that pointed towards exchange processes. The fairly broad width in combination with the exchange features indicated an altered mobility of the spins compared to free Brownian motion, as observed in many molecular liquids. Self-aggregation of IL cations represents one possible explanation. Although it was found that the fraction of aggregated cations was small for [Pyr_13_], the combination with exchange on the order of or even below milli-seconds may cause an underestimation of the fraction of aggregated species [68]. 

The neat [Pyr_16_][Tf_2_N] showed a faster *T*_2,app_ mean relaxation of 72 ms, indicating a reduced mobility of the moieties driven by charge or viscosity effects, with [Pyr_16_][Tf_2_N] showing a 60% higher viscosity compared to [Pyr_13_][Tf_2_N] [69]. The values of *T*_2,app_ vary for the different resonances. The resonance at around 0.5 ppm showed a decrease of *T*_2,app_ towards 44 ms, whereas for the signal at about 1.4 ppm, *T*_2,app_ increased to 100 ms. This indicated that the local mobility of each functional group, rather than collective motion of the IL cation, was the dominant dynamic process responsible for transverse relaxation, although a more complex correlated motion also could not be excluded [70]. 

All resonances of the [Pyr_16_] cation showed a frequency-dependent *T*_2,app_ that decreased in an upfield direction. This manifested itself in two superimposed spectra shifted by 0.07 ppm with different *T*_2,app_; hence, mixing between the two configurations occurred on the order of the transverse relaxation rate. Slower mixing compared to [Pyr_13_][Tf_2_N] was also supported by distinctively broader *T*_2,app_ distributions for each resonance of [Pyr_16_][Tf_2_N] compared to [Pyr_13_][Tf_2_N]. The reason for the two species could be a distribution of aggregated and separated [Pyr_16_] cations. The more slowly relaxing downfield shifted spectrum, corresponding to faster dynamics of the respective species, was probably caused by free [Pyr_16_] cations, while the faster relaxing upfield shifted resonances were due to aggregated [Pyr_16_]. The exchange rates were slower than for [Pyr_13_], confirming the hypothesis of stronger, longer-lasting aggregation for [Pyr_16_] [68].

The apolarity of the ILs increased with alkyl chain length. Therefore, [Pyr_16_][Tf_2_N] had a stronger tendency to form micelles and hemi-micelles in bulk solutions compared to [Pyr_13_][Tf_2_N], leading to longer rotational correlation time constants and correspondingly reduced *T*_2,app_. The static samples showed a behavior that can be related to a different aggregation behavior for [Pyr_13_] and [Pyr_16_], which was reported previously [68]. For [Pyr_13_], only weak aggregation with faster exchange was found, pointing at exchange processes that were not fully averaged on the time scale of the echo time, τ_E_ = 200 µs, rather than imperfect shimming as the origin of the line shape asymmetry. 

It was reported that for longer alkyl chain lengths in [Pyr_1*n*_][Tf_2_N] ILs, the interatomic N–N distance decreases, the ion pair molecular volume increases, and the Mulliken charge on atoms slightly decreases. A particularly drastic change in most parameters was found at *n* = 4 [49,68]. Since the N–N distance of the cation and anion decreases for *n* > 4, the ionic couple is packed more densely and the dipolar moment drops, caused by a decrease in charge density on N that leads to decreasing *T*_2,app_ for [Pyr_16_][Tf_2_N] because the denser packing restricts the movement of molecules. The amphiphilic properties of the pyrrolidinium cation can cause self-assembling even in bulk solutions, leading to alkyl chain length-dependent, self-organized structures [68]. Exchange between these meso-domains, micelles, and hemi-micelles can cause smeared *T*_2,app_ distributions. 

If confined to the CB matrix, the overall spectral resolution decreases and the *T*_2,app_ main mode of [Pyr_13_][Tf_2_N] decreases to 3 ms for the *S*_IL_ = 0.2 sample and to 2 ms for the *S*_IL_ = 1 sample. For [Pyr_16_][Tf_2_N], the *T*_2,app_ main mode decreased to 1.3 ms for *S*_IL_ = 0.2 and to 2.5 ms for *S*_IL_ = 1. The ratio of *T*_2,app_ between neat IL and under confinement was on the same order of magnitude for both IL. The *T*_2,app_ distributions for *S*_IL_ = 0.2 [Pyr_13_][Tf_2_N] were in a range of 1 ms to 5 ms, whereas [Pyr_16_][Tf_2_N] showed a distribution of 0.4 ms to 3 ms. This needs to be considered in the discussion of the diffusion measurements, since signal contributions with *T*_2,app_ < 2 ms will be considerably affected by *T*_2_ weighting. For *S*_IL_ = 1, the range of *T*_2,app_ for [Pyr_13_][Tf_2_N] was between 0.6 ms and 4 ms. *T*_2,app_ for *S*_IL_ = 1 [Pyr_16_][Tf_2_N] confined to CB ranges from 0.4 ms to 3 ms with no dependence on chemical shift. The drastic drop in *T*_2,app_ compared to the neat ILs was caused by an ordering of the IL inside the carbon matrix and at the carbon surface equally for both ILs. This behavior might further be driven either by a separation of anion and cation below a critical pore diameter or slit distance, or by changes in conformation of the [Tf_2_N] anion between cisoid (cis) and transoid (trans) and, thus, a change in Coulombic forces between [Pyr_1*n*_]^+^ and [Tf_2_N]^−^ [71]. 

The *T*_2,app_ of the two ILs approached a similar value at high IL loading. A possible explanation could be an increased exchange between all different pore space environments at increased pore space loading. If the ILs are preferably located on the CB surface due to its high ionophilicity, a higher degree of pore space filling leads, on average, to a reduced pathway length for exchange between different sample environments. An analogous hypothesis was phrased previously based on NMR experiments on static samples [59].

The weighted residuals did not show systematic features, yet this may have been reinforced by the approximation procedure used here, where the weighting matrix was determined iteratively. While underregularization was avoided with this procedure, overregularization may occur in situations where systematic residuals are present due to the unsuitability of the exponential kernel for certain exchange features or oscillations in the echo amplitude evolution [59]. A more accurate method that provides more faithful results would be an independent noise determination based on multiple repetitions of the experiment, followed by a noise analysis for each data point [59]. Since this was an initial study establishing the general applicability of the experimental protocol for this class of multi-component materials, noise analysis was deferred to future research.

Consistent with the shorter *T*_2,app_, the decrease in spectral resolution was more pronounced for [Pyr_16_][Tf_2_N] than for [Pyr_13_][Tf_2_N], which might have been caused by the difference in viscosity and a possible change in cis-trans conformation of [Tf_2_N]^−^ that, in turn, affected [Pyr_1*n*_]^+^. Furthermore, restricted geometries can have significant effects on second-order or kinetic phase transitions, e.g., glass transition temperature, which can lead to a semi-solidification of the IL and, thus, coincide with a decrease in *T*_2,app_. Since the line broadening was mainly a *T*_2_ effect, MAS NMR can be considered successful for the investigated samples. 

The experiments of IL loaded into CB showed a pronounced loading fraction dependence. For both ILs at low pore space loading, two spectral signatures with different *T*_2,app_ could be distinguished. While both had the general features of the respective neat IL, one showed a considerable relaxation broadening and an upfield shift of about 0.5 ppm, while the other corresponded approximately to the spectrum of the neat IL, although with a different relaxation behavior that can only be explained by exchange. The fast relaxing, broad feature was strongly dominant in terms of intensity, with an integral that was about two orders of magnitude higher. Its linewidth could be fully explained by *T*_2_ relaxation. No residual anisotropic interactions or distributions of chemical shifts were necessary. Such a feature was explained in the past by near-surface IL with restricted mobility [45,46,48]. The absence of a considerable chemical shift distribution indicated that different environments that may contribute to this signal were sampled by the ions on a time scale that was fast compared to the measured *T*_2,app_. The neat-like IL signal in CB was not observed before in static NMR experiments. It was, however, similar to a feature that was observed at a particular IL loading level, with sufficient IL to fully cover the CB surface and then left, in addition, some surplus IL that showed a very high mobility on top of this surface layer [59]. Despite a similar loading fraction of the pore space, the looser CB packing in the current work appeared to have prevented the long-range mobility observed in [59]; yet data were consistent with the existence of an additional mobile IL fraction in the partially loaded CB samples as well. 

The observation that the linewidth of the mobile species was narrower than what would be suggested by *T*_2,app._ At least in some parts of the 2D map in Figure 1 there is a strong indication for chemical exchange with the less mobile species. Relaxation and detection of the echo were chronologically separated; hence, narrower signals than suggested by *T*_2,app_ can originate from species that were relaxing in one environment and then exchanged prior to detection into an environment with slower relaxation. The observation of elongated distributions of *T*_2,app_ down to the mode of the main species confirmed such a hypothesis [59]. Such an exchange behavior also indicated that this species was not artificially created by centrifugation due to MAS, but that mobile and less mobile IL species were in close proximity and not separated due to centrifugal forces. When considering the spectrum of the narrow species of the [Pyr_16_] cation, it did not split into two separate species as the neat IL, indicating that within the pore space no aggregates of IL cations were formed. 

An unexpected feature was the increase of the main relaxation mode for [Pyr_16_][Tf_2_N] from *S*_IL_ = 0.2 to *S*_IL_ = 1, while *T*_2,app_ further decreased for [Pyr_13_][Tf_2_N]. When considering the simple model of two environments for the IL, one on the CB surface and one more bulk-like, then an increase of the loading past the level where the CB surface was fully covered (approximately at *S*_IL_ = 0.2) would indicate an increase of an averaged *T*_2,app_, as was observed for [Pyr_16_][Tf_2_N]. In contrast, [Pyr_13_][Tf_2_N] showed a qualitatively different behavior, indicating that this simple model was not valid. An alternative explanation may be suggested by considering a 3D network with ionic channels forming in the neat IL [51]. Such a 3D network is structurally competing with the 2D configuration on the surface of the IL, as discussed above. There will be a transition region in between. Since [Pyr_16_][Tf_2_N] shows a more stable, slower exchanging aggregation behavior in the bulk, its 3D configuration may be more stable. At the same time, [Pyr_13_][Tf_2_N] has a lower viscosity; hence, diffusional motion could cause an enlargement of the transition region. Within the pore network of the CB, the formation of a bulk-like environment with a concomitant increase of a mean *T*_2,app_ may, thus, be prevented for *S*_IL_ = 1 [Pyr_13_][Tf_2_N]. 

This may have far reaching consequences when optimizing IL-based electrolytes, since one of the main aims of IL engineering for electrochemical purposes is to enhance the mobility of the electrolyte and prevent the formation of low-mobility aggregates [59]. The result obtained here suggests that the dynamic properties of such an electrolyte in contact with an electrode cannot be implied based on the properties of the neat electrolyte alone. In addition to modifying the IL structure and the selection of a suitable electrolyte salt, IL electrolytes also offer the possibility for engineering their physical properties by altering the electrode surface. Similar hypotheses have been made based on theoretical considerations [72,73].

### 2.3. Diffusion Measurements

**Figure 2:**
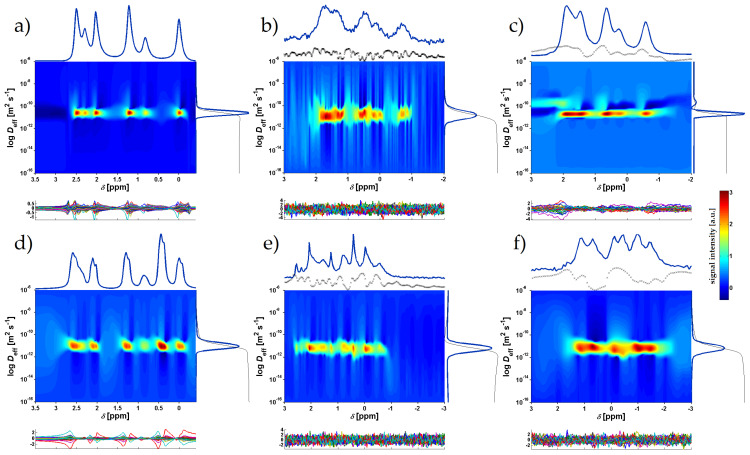
Pseudocolor plots of [Pyr_13_] (**a**–**c**) and [Pyr_16_] (**d**–**f**) diffusion coefficient distributions (Δ = 0.1 s, *δ* = 0.0015 s) vs. ^1^H NMR frequency: (**a**) neat [Pyr_13_][Tf_2_N], (**b**) sample with CB and *S*_IL_ = 0.2 [Pyr_13_][Tf_2_N], (**c**) sample with CB and *S*_IL_ = 1 [Pyr_13_][Tf_2_N], (**d**) neat [Pyr_16_][Tf_2_N], (**e**) sample with CB and *S*_IL_ = 0.2 [Pyr_16_][Tf_2_N], (**f**) sample with CB and *S*_IL_ = 1 [Pyr_16_][Tf_2_N]. The colormap is scaled with the square root of the amplitude to amplify features with low intensity except for panel (**b**), where due to the low signal-to-noise ratio only noise was enhanced by this procedure. The sum projection onto the frequency axis (blue) and the integral of the slowly relaxing regions (dotted black) are shown on top of each panel and the sum projection onto the diffusion axis (blue) and the cumulative sum (black) to its right. The residuals are shown at the bottom of each panel.

The spectrally resolved diffusion data confirmed and refined the *T*_2,app_ data for the different samples. For all samples, exchange features were visible (Figure 2). These were apparent from the negative contributions that could not be removed without creating additional, systematic residuals [74]. On the other hand, only the neat [Pyr_16_][Tf_2_N] sample showed spectrally separable diffusion variations. The upfield shifted spectrum in Figure 2d showed a slightly slower diffusion coefficient of *D*_eff_ = 6.9 × 10^−13^ m^2^ s^−1^ as compared to 8.7 × 10^−13^ m^2^ s^−1^ for the downfield component. Considering the slow exchange between the two environments, as evidenced by the *T*_2,app_ data, such a differentiation was expected with a mixing time in the PFG experiment of Δ = 0.1 s. All the other spectra showed considerably faster exchange (Figure 2a–c,e–f), leading to exchange mixing during the diffusional mixing time Δ and coalescence of the effective diffusion coefficient in a single mode. In addition, the slower diffusion of the upfield-shifted component was consistent with its assignment to aggregated species, as suggested by *T*_2,app_ data.

The observed exchange features differed considerably between the samples. While the *S*_IL_ = 0.2 [Pyr_13_][Tf_2_N] sample (Figure 2b) showed a low signal-to-noise ratio and exchange features were difficult to assess, for *S*_IL_ = 1 [Pyr_13_][Tf_2_N] (Figure 2c) considerable exchange was visible. It showed a downfield-shifted broad feature with a poor spectral resolution despite MAS. Such a species was observed previously with static samples [59] and could be caused by IL in the immediate vicinity of the CB surface near crystallite edges, in slit-shaped cavities, or on amorphous regions [21]. Due to the short *T*_2_ of this feature, only [Pyr_13_] cations that were in a more slowly relaxing environment at the upper end of *T*_2,app_ during encoding realistically contributed to this signal. 

The sample *S*_IL_ = 0.2 [Pyr_16_][Tf_2_N] (Figure 2e) in CB produced spectrally resolved features, yet showed complete mixing of the diffusion coefficient during Δ. Finally, the *S*_IL_ = 1 [Pyr_16_][Tf_2_N] (Figure 2f) in CB sample showed broad exchange features that could not be easily assigned without spectral simulations that considered all the different environments, yet there was only one main mode in the diffusion dimension, also consistent with mixing of the different features during Δ, as already shown by the spectrally resolved CPMG data. 

### 2.4. Technical Aspects

**Figure 3:**
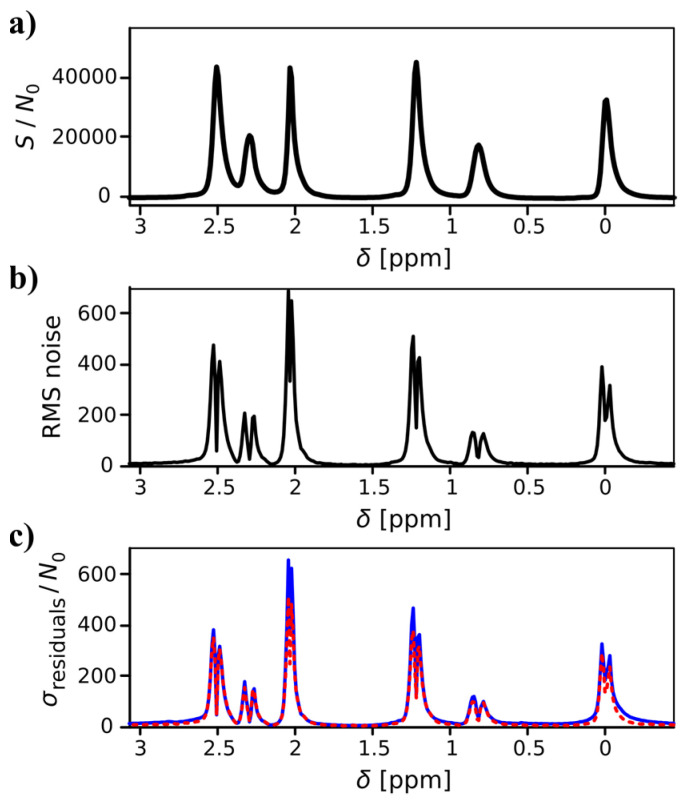
Illustration of multiplicative noise in ^1^H NMR *T*_2,app_ data of neat [Pyr_13_][Tf_2_N]. (**a**) ^1^H NMR spectrum of first echo. The signal intensity is normalized to the noise standard deviation *N*_0_ in the absence of any signal. Notice the very high signal-to-noise ratio, which is a prerequisite for the observation of multiplicative noise. (**b**) Estimation of root-mean-square (RMS) noise level of the above spectrum. The shape is characteristic for multiplicative phase noise, with a maximum at positions with maximum first derivative of the signal [75]. (**c**) Standard deviation of the residuals of the ILT fit (blue) and estimation of the standard deviation based on the assumption of multiplicative phase noise, as depicted in (**b**) (red dashed).

Primarily for the CPMG data but less prominent also for the diffusion experiments, the residuals of the Laplace inversion were considerably higher than the random noise level of the measurements. This observation contrasts the results of our previous study using a standard diffusion probe for static samples, where residuals above the random noise level were only observed for experiments with significant exchange contributions (see supporting info of [59]). In these cases, the systematic residuals could be traced back to features that could not be faithfully reproduced with the kernel chosen for the inversion. In the current case, however, the residuals were significantly higher. In addition, they showed typical features of multiplicative noise [59], as shown for the neat [Pyr_13_][Tf_2_N] sample in Figure 3. When plotting the standard deviation of the residuals vs. frequency, the maximum appears at positions with maximum slope of the resonances, which is characteristic for multiplicative phase noise. In contrast, if features cannot be fitted with a particular kernel or if the inversion was overregularized, then the maximum residuals occur at the peak maxima. Multiplicative noise is not a fundamental problem of the method. The *T*_2,app_ data used for Figure 1a,d was recorded without sample spinning and without pulsed field gradients. Therefore, it was possible to reduce the impact of multiplicative noise for the neat [Pyr_13_] sample to some degree by using adhesive tape to more rigidly fix the MAS rotor in the probe. In principle, it should be possible to obtain the same data quality as in [59] and this work may indicate the scope for future improvements in experimental design and hardware, especially for MAS PFG NMR. Partial remedy is possible by slightly overcoupling the resonator to reduce its quality factor or by working with reduced field gradient strength. While both measures are quite suitable for liquid or IL samples in porous media, it does limit the applicability for the investigation of solid electrolyte materials. In particular for solid Li ion conductors, chemical shift differences are small and high spinning frequencies are desirable for optimum resolution, while at the same time, strong PFGs are necessary to limit their duration and maximize the range of diffusion coefficients that can be distinguished towards low mobility. Therefore, for a wide applicability beyond proof-of-concept work, further development of the experimental method is desirable.

In addition to random multiplicative noise, which was a major concern mainly for the neat IL samples, CPMG data of IL loaded CB samples also showed pronounced, spectrally dependent oscillations of the echo amplitudes with longer periods than the well-known oscillations between even and odd echoes [76]. Fast relaxing components were affected more strongly by such oscillations. The effect can be observed best in the *T*_2,app_ data of the [Pyr_13_][Tf_2_N]-loaded CB samples at the upfield and the downfield ends of the main species, where intensive negative features are observed. The spectral signature of these features suggests an origin that is not mainly dependent on hardware limitations, but has a more fundamental reason. Protons of [Pyr_13_] cations in direct proximity of CB surfaces are more strongly affected. The interaction of the oscillating magnetic field caused by the NMR pulses with the conducting CB surface leads to a local field enhancement or shielding as well as a phase shift. However, the heterogeneity of the CB structure prevents the more long-range formation of eddy currents; therefore, only IL in the immediate vicinity of the surface is affected. The resulting local phase shifts cannot be corrected by phase corrections, as not all of the sample is affected, necessarily leading to negative contributions in spectrally resolved relaxation measurements. The oscillations may provide information about electrical properties of the porous host, suggesting that more in-depth investigations of these effects may be promising. 

For an optimum extraction of available information in data with non-uniform noise, data analysis schemes using weighted data are called for [77,78], as applied here for the CPMG data presented in Figure 1. The oscillating features of the echo decay were also treated as noise, since the exponential kernel used for the Laplace inversion is not suitable to fit oscillations. To extract the full information contained in the data, it may be worthwhile to explore more sophisticated data analysis protocols in the future. 

### 2.5. Influence of Centrifugal Forces

A simple estimate of the centrifugal forces acting on the IL at a MAS spinning rate of 5 kHz and a pore radius where pores remain saturated can be made based on the relationship [19]

(1) $$P=\pi^{2}\nu^{2}r_{r}{}^{2}\rho$$

where *P* is the pressure (Pa), *ν* is the spinning rate (Hz), *r_r_* is the radius of the rotor or insert (m), and *ρ* is the sample density (kg m^−3^). The pressure exerted on the IL for the given experimental parameters is 434 kPa and 467 kPa for [Pyr_16_][Tf_2_N] and [Pyr_13_][Tf_2_N], respectively. A pressure of *P* = 33 kPa can be assumed for the CB (*ρ* ≈ 100 kg m^−3^). For pores with a circular cross-section the corresponding pore radius can be calculated using [79]

(2) $$r_{c}=\frac{2\gamma \cos\sigma }{P}$$

where *σ* is the contact angle and *γ* the surface tension of 0.0302 J m^−2^ and 0.0353 J m^−2^ for [Pyr_16_][Tf_2_N] and [Pyr_13_][Tf_2_N], respectively [80,81,82]. Assuming a good wetting of the carbon surface by the ILs with *σ* < 60° yields *r_c_* > 69 nm for [Pyr_16_][Tf_2_N] and *r_c_* > 75 nm for [Pyr_13_][Tf_2_N]. Since pore size distributions from *r* = 1 nm to 60 nm have been reported for Vulcan^®^ carbon black XC 72 and XC 72R [83,84,85,86,87,88], a centrifugal effect on the ILs is not expected because *r_c_* > *r*. 

### 2.6. Influence of Sample Preparation and NMR Parameters on MAS Measurements

Since CBs can be employed either as insulating or conductive additives depending on the density, both the filling fraction of CB pore space and the exerted pressure on the CB during rotor loading determine the electrical properties of the system. Electrical percolation in CBs is a known phenomenon, where a certain electrical percolation threshold determines the formation of π-electron conducting networks [89]. By increasing the graphitic character of the surface, the electrical conductivity of CBs increases, whereas for CBs possessing a low surface area, the electrical conductivity correlates with the surface chemistry [90]. For Vulcan^®^ carbon black XC 72, the electrical conductivity increases linearly up to 2.7 S cm^−1^ at a pressure of 2000 kPa [90]. While spinning, the centrifugal forces will cause an additional compaction of the sample and, if the sample is a solid/liquid mixture, an electrically conductive slurry originates. 

The sample rotation in the external magnetic field *B*_0_ can cause eddy currents, which can slow down the rotor speed [91]. Therefore, filling and compacting CB into MAS rotors is the first delicate step that can impact the tuning and matching of the system to a point where the sample becomes immeasurable. For the system under study, an increase in the CB electrical conductivity to *σ* ≈ 0.43 S cm^−1^ can be assumed for a spinning rate of 5 kHz. The electrical conductivity of the ILs is negligible at 0.0015 S cm^−1^ and 0.005 S cm^−1^ for [Pyr_16_][Tf_2_N] and [Pyr_13_][Tf_2_N], respectively. Thus, the compaction pressure exerted on the CB during sample preparation determines the conductive properties of the systems, whereas the increase in conductivity for the applied CB can be neglected for a spinning rate of 5 kHz.

CB samples are largely heterogeneous, based on their intrinsic chemical structure and their preparation. Therefore, chemically identical nuclei can experience different local environments and, in addition to anisotropic magnetic susceptibility that can be averaged out by MAS, variations of isotropic chemical shifts or local susceptibilities throughout the sample may occur, which can cause signal broadening.

Adding liquid to a porous, solid matrix will cause the formation of air bubbles and trapped air in dead pores (unconnected pores). These air pockets can decrease the spectral resolution and additionally alter the magnetic susceptibility of the samples. Therefore, an accurate filling and saturation procedure is imperative for proper MAS diffusion measurements.

Another source that can possibly alter diffusion measurements is the Lorentz force exerted on charges due to rapidly switched electrical currents in the gradient coils, possibly causing phase shifts, additional signal attenuation, artificial diffraction patterns in the diffusion data, loss of tuning and matching, or even a rotor crash [91,92,93]. The force and the resulting vibrations are a function of the applied current, the gradient wave form, its duration and its rise and fall time, the magnetic field, and the density of coil turns. The vibrations cause further phase shifts in the resulting NMR spectra, which can lead to artificial features in the ILT data. For the system under study, limiting the gradient strength to half maximum eliminated vibrational effects on the sample.

## 3. Materials and Methods

### 3.1. Sample Preparation and Characterization

The 1-methyl-1-propylpyrrolidiniumbis(trifluoromethylsulfonyl)imide ([Pyr_13_][Tf_2_N]) (water content < 100 ppm, density = 1.42 g cm^−3^ at 303.15K, melting point = 255.15K) and 1-hexyl-1-methylpyrrolidiniumbis(trifluoromethylsulfonyl)imide ([Pyr_16_][Tf_2_N]) (water content <100 ppm, density = 1.32 g cm^−3^ at 293.15K, melting point = 278.15K) were both purchased from Iolitec (Germany) and used as received (values taken from [69,94]). Both ILs were degassed under vacuum for 24 h prior to measurements. 

The neat [Pyr_16_][Tf_2_N] and [Pyr_13_][Tf_2_N] were loaded into 30 µL, 4 mm HR-MAS cylindrical disposable inserts (Bruker, Germany) with an inner radius of 1 mm using a micro syringe (for details on the insert, see [93]). Each insert was sealed with a cap and screw and placed inside a cylindrical 4 mm MAS rotor (Bruker, Leipzig, Germany).

Vulcan^®^ carbon black XC 72 (furnace black) powder (Cabot Corporation, Boston, MA, USA) with a BET (N_2_) surface area of *A*_BET_ = 218 m^2^ g^−1^, primary particle size of 30 nm, and a density of 264 kg m^−3^ was grinded using an agate mortar. Since grinding alters the bead shape of Vulcan^®^ XC72, the resulting CB after grinding is comparable to the powdered Vulcan^®^ XC72R with an average particle size of 50 nm, a density of 96 kg m^−3^, and *A*_BET_ = 222 m^2^ g^−1^. A more detailed description of the physical properties of Vulcan^®^ XC72 and XC72R is given in [83,84,85,86,87,88,95,96]. A total pore volume of *V*_total_ = 0.76 cm^3^ g^−1^ was determined by N_2_ adsorption using an Autosorb iQ 2 (Quantachrome, Boynton Beach, FL, USA) equipped with a cryocooler (CTI-cryogenics, Waltham, MA, USA). A mean pore radius of $\overset{¯}{r}$ = 7 nm was estimated using the relationship 2*V*_total_/*A*_BET_. 

Approximately 6 mg of grinded CB was loaded and gently compacted into the 30 µL disposable HR-MAS insert, achieving a bulk density of ≈0.2 g cm^−3^, assuming a particle density of 1.8 g cm^−3^.

ILs were added to the CB using a microliter syringe (Hamilton, Reno, NV, USA). For each of the two ILs, two different IL volumes were applied to fill a certain fraction of the CB pore space. Since CB was filled into the MAS rotor in the form of a packed bed rather than pressed to a pellet, as in our previous work [59], the exact volume fraction was difficult to determine. As a rough estimate, about 20% of the pore space was filled in one set of samples (IL saturation level *S*_IL_ = 0.2) and the full pore space in the other set (*S*_IL_ = 1), while care had been taken that no free IL was present, as confirmed by the absence of corresponding features in the NMR data. The IL was applied dropwise, achieving the different saturation levels of *S*_IL_ = 0.2 (1 drop) and *S*_IL_ = 1 (5 drops) both for [Pyr_16_][Tf_2_N] and [Pyr_13_][Tf_2_N]. Afterwards the insert was placed under vacuum for 1 h using a vacuum desiccator until all ionic liquid was absorbed by the CB. The mass added was determined by means of NMR saturation profiles, yielding an amount of 1.84 mg (0.37mg/drop) [Pyr_16_][Tf_2_N] and 1.04 mg (0.21mg/drop) [Pyr_13_][Tf_2_N] for the fully saturated samples. 

### 3.2. ^1^H-NMR Measurements

All ^1^H-NMR MAS experiments were performed using a 9.4T Bruker Avance spectrometer with a 1.5 T m^−1^ micro 2.5 imaging gradient system, equipped with a Bruker 4-mm ^1^H/^13^C HR-MAS probe. A probe temperature of 303.15 K was maintained for all measurements. The apparent transverse relaxation time constants (*T*_2,app_) were monitored using the Carr-Purcell-Meiboom-Gill (CPMG) method [97]. For diffusion measurements, a 13-interval, stimulated echo PGSTEBP Bruker pulse sequence was employed, as described in [98,99] following the approach of Stejskal and Tanner [13] using a gradient pulse duration of *δ* = 0.0021 s, observation time of Δ = 0.1 s, 32 gradient steps, *T*_1_ relaxation delay RD = 2.5 s, and a radio frequency pulse duration of 0.006 ms for 90° pulses. Measurements of the neat ILs were performed under static conditions, whereas for ILs confined to CB a MAS spinning speed of 5 kHz was applied. 

No external or internal reference compound was employed for all measurements. Analogously to [100], the ^1^H peak position of the terminal alkyl group protons served as 0 ppm reference for [Pyr_16_][Tf_2_N] and [Pyr_13_][Tf_2_N].

### 3.3. Data Analysis

For the 13-interval PGSTE method, the attenuation of the echo amplitude is given by [99]

(3) $$\ln\frac{S\left(g_{a}\right)}{S\left(0\right)}=-\gamma^{2}D[\delta^{2}\left(4\Delta +6\tau -\frac{2}{3}\delta \right)g_{a}^{2}+2\tau \delta \left(\delta_{1}-\delta_{2}\right)g_{a}g_{0}+\frac{4}{3}\tau^{3}g_{0}^{2}]$$

where *S* is the signal intensity, *γ* is the gyromagnetic ratio (2.6751 × 10^8^ rad s^−1^ T^−1^ for ^1^H), *g*_0_ is the background (internal) gradient, *g*_a_ is the applied gradient, *δ* is the duration of the applied gradient, $\delta_{1}$ is the time interval between the first rf-pulse and the start of *g*_a_, and $\delta_{2}$ is the time interval following the end of *g_a_* until the second rf-pulse, where for all measurements *δ*_1_ = *δ*_2_. Since *δ*_1_ *=* *δ*_2_, the second term in Equation (3) disappears while the third term can be eliminated by normalizing the signal with pulsed gradients applied with the signal *S*(*g_a_* = 0) = S(0) without pulsed gradients. Δ is the interval between the second and the third gradient pulses, *τ* is the time between the rf-pulses, and *D* is the self-diffusion coefficient [101,102,103]. 

Both the 13-interval PGSTE and the CPMG data were spectrally resolved by Fourier transforming the NMR raw data along the transient dimension. The distributions of *T*_2,app_ and *D_eff_* were obtained by performing a regularized inversion with an exponential kernel without a non-negativity constraint [75,104]. Parameterization was done as described in [78] without manually fine-tuning the parameters. 

CPMG experiments showed initial oscillations of the echo amplitude due to pulse imperfections and off-resonance effects, which are partially caused by the electrically conductive porous CB matrix that induces a distribution of the amplitude, *B*_1_, of the oscillating radio frequency field used to excite the spins. This effect was reduced by using only even echoes for data processing [76]. In addition, slower oscillations of the echo amplitudes were apparent as well, which caused problems with parametrization of the regularized inversion. To avoid overregularization of data points that were not affected by these oscillations, the oscillations were considered as noise and data were weighted accordingly [77]. Since, as detailed in the discussion, data were also affected by multiplicative noise, the data weighting was determined iteratively [75]. First, using the residuals of an initial inversion with unweighted data, smoothed over three echoes, an estimate for the weight matrix was obtained. This procedure was repeated once using the residuals of an inversion with weighted data. Further iterations only led to marginal additional changes. For the PGSTE data, due to the lower signal-to-noise ratio that reduced the impact of multiplicative noise and the constant timing of the experiment that prevented oscillations from pulse imperfections, the sensitivity was mostly limited by additive white noise, and inversion was done using unweighted data.

In the figures, unless stated otherwise, the *T*_2,app_ and the *D_eff_* coefficient distributions are represented as square root scaled color maps with conservation of the sign to enhance weaker spectral features.

## 4. Conclusions

We investigated the suitability of MAS NMR with and without PFG for the investigation of ionic liquids in electrically conductive porous CB as a model system for an IL-based electrolyte in contact with a porous electrode. Using CPMG and diffusion experiments, it was possible to achieve spectral resolutions consistent with apparent *T*_2_ relaxation time constants. The increased resolution afforded by MAS allowed the identification of weak, narrow spectral features of the investigated IL at partial loading of the CB host. These were caused by IL cations with a high local mobility; yet fast exchange indicated that a spatially distributed species was responsible for these features rather than a reservoir created by centrifugal force exerted by MAS sample spinning.

When comparing the local mobility of neat IL with IL in CB, significant differences were observed. In particular, the relaxation and the associated spectral properties varied to an extent that suggested that bulk IL studies were ill-suited for the optimization of electrolyte properties in a porous electrode environment. A porous host system may alter the aggregation behavior of ILs and thereby provide an additional degree of freedom to optimize transport in IL electrolyte-based electrochemical systems. One example is the existence of highly mobile IL on top of a more strongly bound surface layer, as suggested in earlier work [59] and supported here by evidence of exchange. 

While overall the samples showed a very good signal-to-noise ratio, the signal was affected by multiplicative noise. Part of the problem may have been caused by the ionic and conductive nature of the sample, with ions interacting with electromagnetic fields and an electrically conductive porous host, leading to partial shielding of the sample. This led to a stronger than usual inhomogeneity of the exciting radio frequency field amplitude *B*_1_ across the sample, causing oscillations of the echo amplitude that are usually common for imperfectly adjusted pulse lengths. For the data analysis, these oscillations were treated as noise, which allowed an estimate of the sensitivity for individual data points and, consequently, Laplace inversion with weighted data. Thereby, signal contributions with very fast relaxation were suppressed since no reliable differentiation between fast relaxing and oscillating features was possible within the scope of this study. While artifacts and spurious signals could be avoided, there may have been some information lost with regard to signal components that relax during the course of a small number of echoes. A more refined analysis appears possible, warranting future research while extending the potentials of MAS PFG NMR for this class of materials.

MAS NMR facilitated an improved resolution of IL spectra in porous CB, which allowed the identification of features so far not reported in literature. However, an exact identification of the different environments is still challenging due to the hierarchical nature of the CB structure or, more generally, of many porous electrode systems used for electrochemical experiments. Therefore, the investigated techniques provide complementary information rather than substituting established methods.

## Data Availability

The data presented in this study are available on request from the corresponding author. The data are not publicly available due to non-standard, proprietary formatting, which will necessitate explanation on sharing.

## Sample Availability

Samples of IL and CB compounds are available from the authors.
